# Building “walls” to stop pathogens: neutrophils play a role in the repair of extracellular matrix

**DOI:** 10.1186/s40779-025-00624-0

**Published:** 2025-07-10

**Authors:** Feng-Ying Liao, Zhen Wang, Jian-Xin Jiang, Ling Zeng

**Affiliations:** State Key Laboratory of Trauma and Chemical Poisoning, Daping Hospital, Army Medical University, Chongqing, 400042 China

**Keywords:** Matrix-producing neutrophils, Circadian regulation, Skin barrier function, Wound healing, TGF-β signaling

Neutrophils are the first immune responders to infection. They rapidly migrate to the site of infection and phagocytose pathogens. Additionally, they release neutrophil extracellular traps (NETs) to immobilize and kill pathogens via a unique form of cell death known as netosis [1, 2]. Recently, a groundbreaking study published in *Nature* reported that a subgroup of neutrophils contributes to the composition and structure of the extracellular matrix (ECM), reinforces its mechanical properties, and promotes its barrier function in both naive and wounded skin [3]. The structural and functional contributions of these neutrophils support those of “professional” ECM producers (e.g., fibroblasts) in a non-redundant manner by locally modulating the dynamics of the ECM in the skin.

Traditionally, the mechanisms utilized to defend against pathogens have been divided into two groups: structural barriers formed by epithelial and mesenchymal cell networks and biochemical processes (e.g., phagocytosis and cytotoxicity) mediated by innate immune cells. However, the research conducted by Vicanolo et al. [3] challenges this division by demonstrating that neutrophils reinforce the skin barrier. In their study, a collagen type III alpha 1 positive (COL3A1^+^) neutrophil subpopulation exhibited dual functionality in cutaneous defense. In addition to their established immunological roles, COL3A1^+^ neutrophils were found to actively participate in ECM remodeling in murine and human dermal tissues via the activation of transforming growth factor-β (TGF-β) dependent biosynthetic pathways typically associated with mesenchymal cells. The authors concluded that neutrophil involvement in wound healing can be classified into two stages. Within one day after injury, lymphocyte antigen 6 complex, locus G positive (Ly6G^+^) neutrophils clear debris and trigger inflammation. Then, 2 − 3 d after injury, TGF-β-activated COL3A1^+^ neutrophils surround the wound and deposit a 1 mm diameter ECM ring or “wall” rich in COL3A1. Hence, the functions of neutrophils are being redefined as evidence emerges of their capacity to contribute to ECM building and maintenance.

Other studies have also shown that innate immune cells are multifunctional barrier architects. For example, in addition to their classical containment roles, macrophages coordinate fibrin-based bacterial entrapment and perivascular collagen modulation, whereas neutrophils contribute to ECM biosynthesis through collagen type I alpha 1 (COL1A1) deposition in fibrosis [4] and transport ECM components during tissue repair [5]. Interestingly, the ECM-producing activity of neutrophils exhibits circadian regulation, with increased migration and deposition occurring at night, potentially optimizing barrier repair during periods of reduced immune activity [3]. This temporal dimension adds another layer of complexity to neutrophil function, highlighting its dynamic adaptation to the changing needs of the host (Fig. 1).

**Fig. 1:**
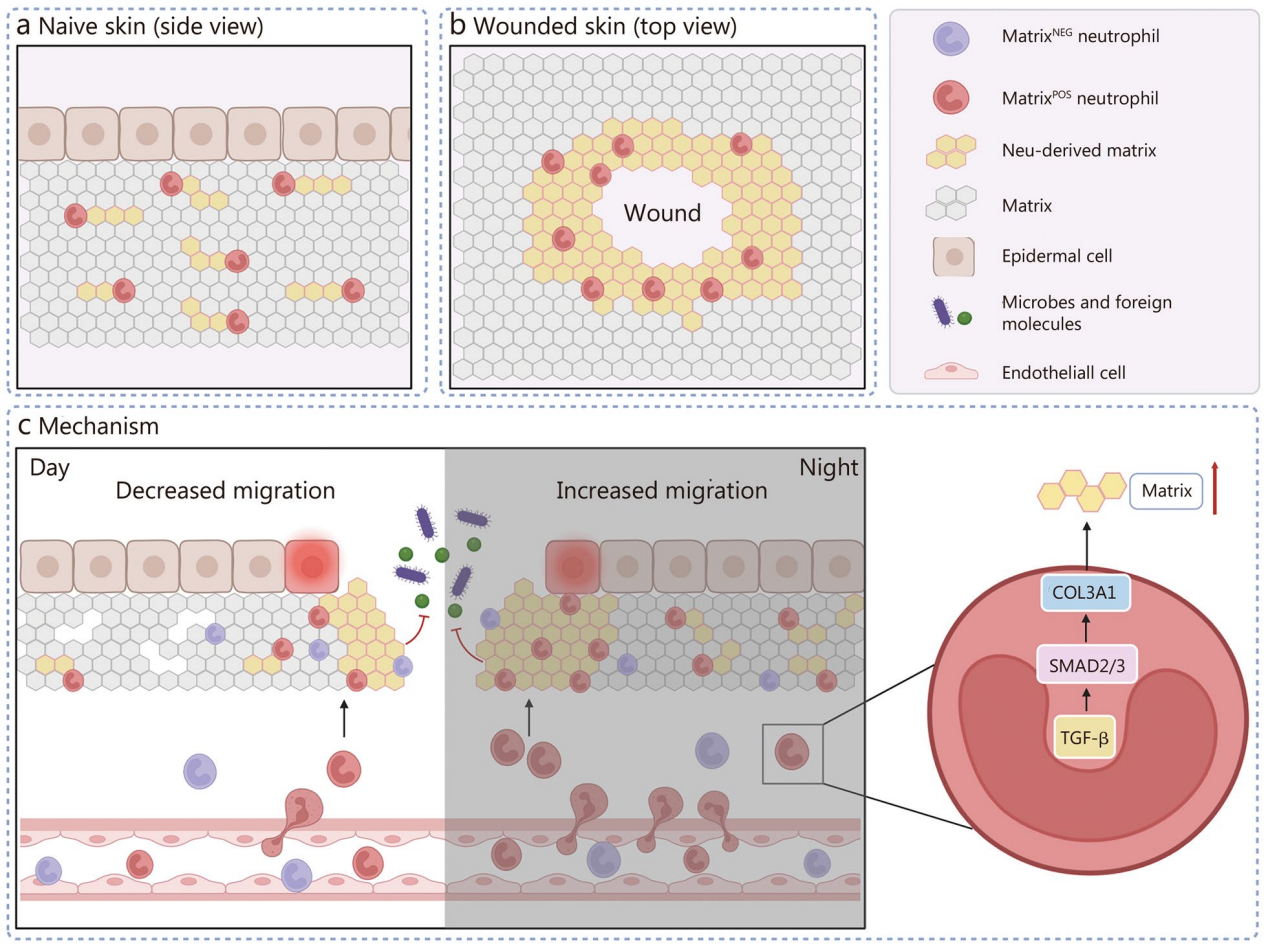
Role of extracellular matrix (ECM)-producing neutrophils in skin barrier maintenance. **a** ECM-producing neutrophils contribute to the composition and structure of the ECM, reinforce its mechanical properties, and promote its barrier function in naive skin. **b** ECM-producing neutrophils build ECM “rings” around wounds, which prevent pathogens from entering wounded skin. **c** Mechanistic depiction of the circadian regulation of neutrophil migration. Migration is lower during the day and higher at night, and it is mediated by signaling through SMAD2/3 and COL3A1. TGF-β transforming growth factor-β, SMAD2/3 mothers against decapentaplegic homolog 2/3, COL3A1 collagen type III alpha 1, Neu neutrophil

Another recent study revealed that the choroid plexus is a dynamic immune organ within the brain that actively orchestrates immune cell recruitment and barrier repair during neuroinflammation [6]. These findings, together with those showing that neutrophils and macrophages have both antimicrobial and barrier maintenance functions, suggest a common theme of immune cells actively contributing to tissue homeostasis and barrier defense across diverse organ systems.

The work of Vicanolo et al. [3] extends the functional spectrum of neutrophils to cutaneous barrier maintenance, in which they exhibit spatiotemporal specialization in ECM remodeling, dynamic regulation of collagen fiber architecture, circadian mechanical properties, and wound-closure kinetics. Crucially, the migratory capacity of neutrophils enables them to perform targeted regulation of ECM microdomains inaccessible to fibroblasts and to act in cooperation with structural cells. Hence, neutrophils can be considered kinetic modulators of barrier integrity due to their capacity to produce ECM components, transport pre-assembled components, and reshape the ECM. The above-mentioned findings underscore the evolutionary ingenuity of utilizing migratory immune cells for structural reinforcement, a strategy that minimizes collateral damage while maximizing barrier resilience.

A multidisciplinary approach involving both immunology and biomechanics is required to elucidate the TGF-β-dependent processes used by neutrophils to build and repair the ECM. Future studies exploring neutrophil-ECM interactions in disease contexts could reveal novel therapeutic avenues for conditions ranging from impaired wound healing to autoimmune fibrosis. Further research is also required to determine whether ECM-producing neutrophils secrete matrix metalloproteinases (MMPs) to balance remodeling, given that proteomic analyses have revealed that MMP8 and MMP9 are expressed in wound exudates, suggesting dual roles for these proteins. It is also necessary to determine the capacity of neutrophils present in other tissue types to support barrier maintenance. For example, lung neutrophils showed weaker ECM signatures; however, atomic force microscopy revealed reduced lung stiffness in mice with neutrophil-specific defects in TGF-β signaling, suggesting broader effects on mucosal barriers.

## Data Availability

Not applicable.

## Abbreviations

COL1A1: Collagen type I alpha 1
COL3A1: Collagen type III alpha 1
ECM: Extracellular matrix
Ly6G: Lymphocyte antigen 6 complex, locus G
MMPs: Matrix metalloproteinases
NETs: Neutrophil extracellular traps
TGF-β: Transforming growth factor-β
